# NOVEL APPROACHES TO OBESITY TREATMENT IN OLDER ADULTS

**DOI:** 10.1093/geroni/igae098.1051

**Published:** 2024-12-31

**Authors:** Barbara Nicklas, Denise Houston, John Batsis

**Affiliations:** Chair; Co-Chair; Discussant

## Abstract

The prevalence of obesity (BMI ≥30 kg/m2) among older adults (65+ years) in the U.S. has almost doubled over the last three decades, with approximately 40% of older adults living with obesity. Lifestyle modifications of moderate caloric restriction have been shown to improve the metabolic and functional health in older adults (e.g., lower blood pressure, fasting glucose, and interleukin-6, and improvements in cardiorespiratory fitness and gait speed). While lifestyle modifications of caloric restriction typically only produce modest weight loss – in the range of 5 to 10% - recent FDA approved medications to treat obesity produce significant weight loss – in the range of 15 to 20%. However, trials of anti-obesity medications have included few older adults. Furthermore, caloric restriction – by either lifestyle modification or anti-obesity medications – results in the loss of muscle and bone, potentially exacerbating sarcopenia and osteopenia. As a result, health care providers are often reluctant to recommend weight loss in older adults with obesity due to concerns regarding the overall safety and benefits of caloric restriction. Thus, different approaches to caloric restriction may be needed to minimize the potential risks of caloric restriction in older adults. This symposium will address the following in older adults: 1) whether time restricted eating (TRE) may be an alternative to caloric restriction (Houston and colleagues); 2) whether replacing lost weight externally reduces weight loss associated musculoskeletal tissue loss (Beavers and colleagues); and 3) whether semaglutide improves physical function, muscle mass, and biomarkers of aging (Cortes and colleagues). Obesity and Aging Interest Group Sponsored Symposium

